# Atomic partial charge predictions for furanoses by random forest regression with atom type symmetry function

**DOI:** 10.1039/c9ra09337k

**Published:** 2020-01-02

**Authors:** Xiaocong Wang, Jun Gao

**Affiliations:** Hubei Key Laboratory of Agricultural Bioinformatics, College of Informatics, Huazhong Agricultural University Wuhan China gaojun@mail.hzau.edu.cn

## Abstract

Furanoses that are components for many important biomolecules have complicated conformational spaces due to the flexible ring and *exo*-cyclic moieties. Machine learning algorithms, which require descriptors as structural inputs, can be used to efficiently compute conformational adaptive (CA) charges to capture the electrostatic potential variations caused by the conformational changes in the molecular mechanics (MM) calculations. In the present study, we introduced atom type symmetry function (ATSF) developed based on atom centered symmetry function (ACSF) for describing conformations for furanoses, in which atoms were categorized by atom types defined by their properties or connectivity in classic molecular mechanics (MM) force field parameters to generate a suitable coordinate size. Random forest regression (RFR) models with ATSF showed improvements for predicting CA charges and dipole moments for furanoses compared to those with ACSF and atom name symmetry functions where atoms were categorized by their unique atom names. The CA charges predicted by RFR models with ATSF showed more comparable reproductions of the carbohydrate–water and carbohydrate–protein interactions computed with RESP charges individually derived from QM calculations than the ensemble-averaged atomic charge sets commonly employed in molecular mechanics force fields, suggesting that the predicted CA charges were capable of including electrostatic variations in their dynamic charge values. Improvements by ATSF showed that categorizing atoms by atom types introduced chemical structural perceptions to descriptors and produced a suitable coordinate size in ATSF to capture key structural features for furanoses. This categorizing scheme also allows ATSF to be readily adopted by other biomolecules thanks to the broad implementations of MM force fields.

## Abstract 2

Atom type symmetry function that utilizes atom types defined in traditional force fields demonstrated improvements for describing structures of furanoses, and the capability of predicting their conformational adaptive charges with random forest regression models.

## Graphical abstract

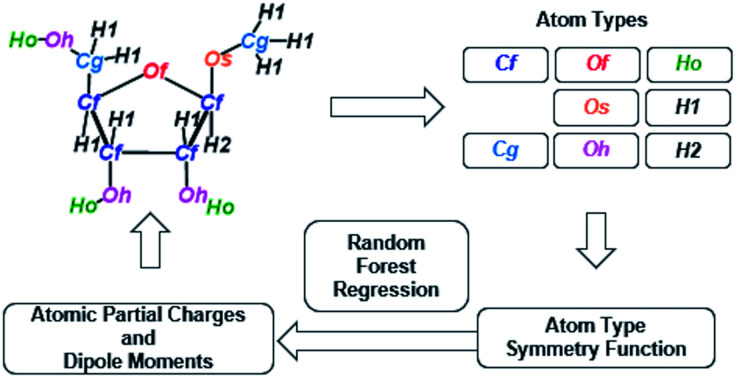

## Introduction

Furanoses are essential components for the backbones of nucleic acids and complex polysaccharides frequently found in organisms ranging from bacteria to protozoa, fungi to plants.^1^ They have complicated conformational spaces as their five-membered ring can adopt multiple stable conformations in addition to the spinning of their abundant *exo*-cyclic groups in solution.^2^ These conformational variations lead to heterogeneous intramolecular properties, such as electrostatic potentials, which affect their recognitions and interactions with other biomolecules.^3^ These variations also made it difficult for classical molecular mechanics (MM) force fields to adequately represent the electrostatic properties for furanoses, as static atomic partial charge models are commonly employed.^4^ These models, however computationally efficient, lack the accuracy to represent the intrinsic electrostatic potential variations. Efforts have been devoted to develop charge models that are capable of adapting conformational variations.^4–10^ Approaches have been proposed and developed, yet, adoptions of these approaches depend on their applicability and ease of use. It is also unfeasible to derive conformational adaptive (CA) charges during MM calculations directly from electrostatic potentials obtained from resource-hogging quantum mechanics (QM) calculations.^5,11,12^ Therefore, it is desirable to efficiently compute QM-quality CA charges that can be used within the classical MM framework.

Machine learning algorithms have been implemented in calculating electrostatic potentials and provide a promising alternative approach for computing CA charges.^13–15^ The accuracy and efficiency of machine learning algorithms critically depend on the descriptors that are used to represent molecular structures.^16,17^ Descriptors for machine learning algorithms, unlike cartesian coordinates, are required to be invariant under permutations of atoms, as well as translations or rotations of the molecule, so as to represent any conformation in a unique set of coordinates.^16^ These descriptors also need to describe the key structural features of molecules with a sufficient size of coordinates. The atom centered symmetry function^18^ (ACSF) introduced by Behler and Parrinello in 2007 has become a prominent descriptor for machine learning algorithms and many successful implementations have been reported.^19–24^ ACSF categorizes atoms by the number of atom types in the molecule, which determines the size of its coordinates. Thus, when describing furanoses and other biomolecules that usually possess complicated conformational spaces but limited types of chemical elements, improvements may be needed.

**Fig. 1:**
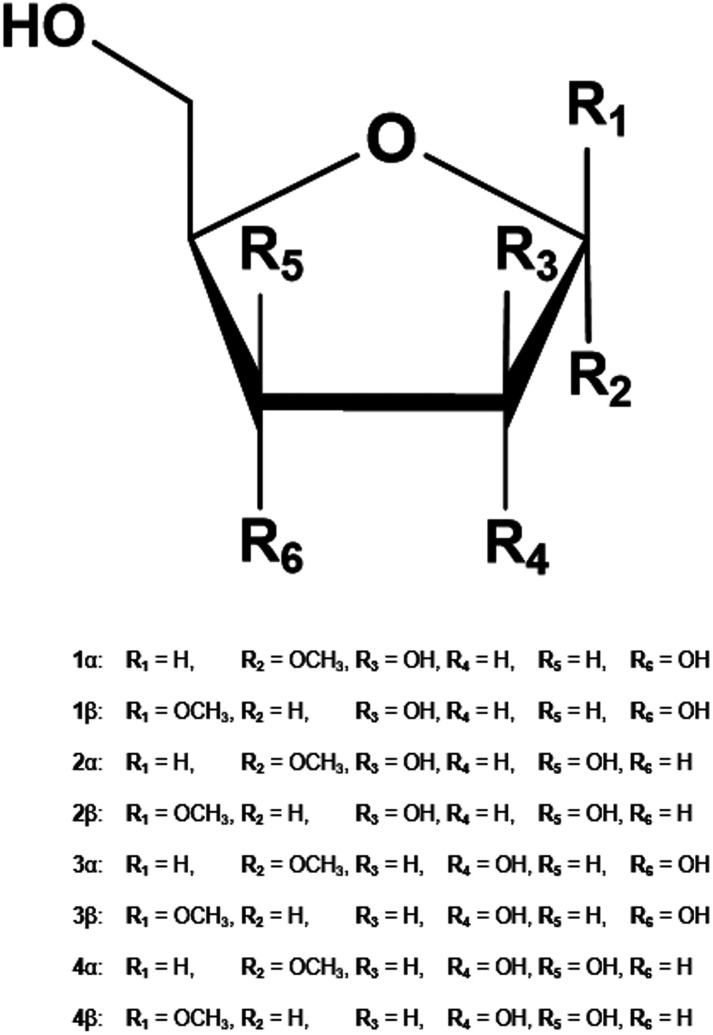
Methyl furanosides for the present study: methyl d-arabinofuranoside (1), methyl d-lyxofuranoside (2), methyl d-ribofuranoside (3), and methyl d-xylofuranoside (4).

In the present study, we introduced atom type symmetry function (ATSF), that categorized atoms by their atom types defined in MM force fields^3,25–27^ and provided more detailed structural descriptions, to predict CA charges with properly trained random forest regression (RFR) models.^28^ Atom type is a well-established and crucial concept embedded in common MM force fields, in which atoms are categorized beyond chemical elements and by their properties or connectivity. In the furanose-specific GLYCAM force field,^3^ atoms for furanoses (Fig. 1) that belong to three chemical elements were further categorized into eight different atom types and the size of coordinates for ATSF increased by more than three times comparing to that for ACSF. The Pearson correlation coefficients for predicted charge values by RFR models with ATSF with reference to RESP charges derived from QM calculations were all above 0.9 and increased averagely by 9% and 4%, respectively, comparing to ACSF and atom name symmetry functions (ANSF). In addition, the predictions of dipole moments were improved by 43% and 48% by charges predicted with ATSF comparing to those with ACSF and ANSF, respectively. Furthermore, the electrostatic related interactions for furanosides, carbohydrate–water and carbohydrate–protein interactions, computed with the CA charges predicted by RFR models with ATSF reduced the average error by more than half for that calculated with the static ensemble-averaged charge models and individual RESP charges derived from QM calculations, which indicated that the predicted CA charges was capable of including electrostatic variations in their dynamic charge values.

## Methods

### Training and testing dataset formation

**Fig. 2:**
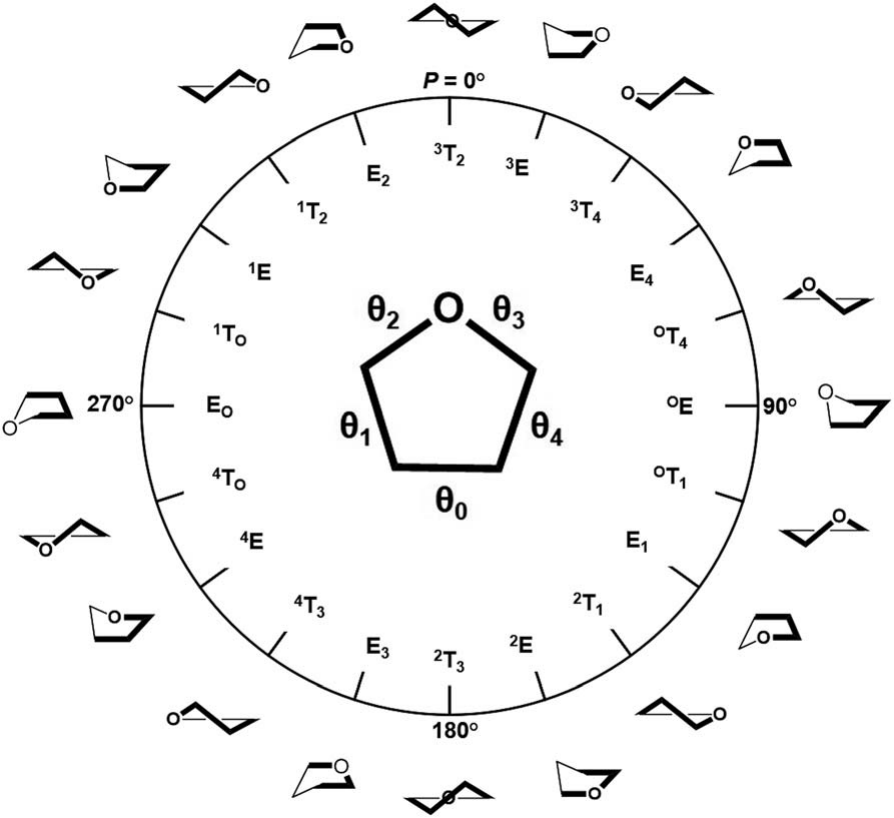
Pseudorotational itinerary of furanoses depicting different Envelope (E) and Twist (T) ring conformations with associated conformational phase angle (*P*) values in degrees. The inset demonstrated the definitions of five ring torsion angles: *θ*_0_ = C1–C2–C3–C4, *θ*_1_ = C2–C3–C4–O4, *θ*_2_ = C3–C4–O4–C1, *θ*_3_ = C4–O4–C1–C2, and *θ*_4_ = O4–C1–C2–C3.

The training and testing datasets for RFR models were formed by combining the samplings for the *endo*- and *exo*-cyclic conformations for furanosides, in order to sufficiently cover their conformational spaces. The conformations for the five-membered ring are determined by the *endo*-cyclic rotations and can be described by the pseudorotational itinerary,^29,30^ where the phase angle (*P*) and puckering amplitude (*τ*_m_) can be calculated by the five ring torsion angles (Fig. 2).1
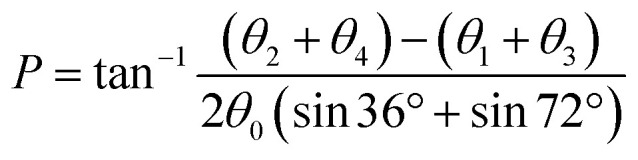
2
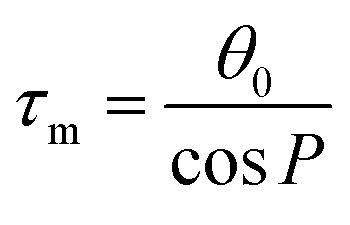


In order to thoroughly sample the conformational spaces for the furanose ring, *τ*_m_ was iterated from 3 to 45° at a 3° interval, and *P* was from 0 to 360° at a 6° interval. A total of 900 ring conformations were generated for each furanoside or furanose. Unlike the intertwining *endo*-cyclic torsions, all *exo*-cyclic rotations were independent from each other, so it is hardly to systematically cover *exo*-cyclic conformational spaces. So, 30 combinations of randomly assigned values for all *exo*-cyclic dihedral angles were created for each ring conformation, in which 24 structures were randomly selected to construct the training dataset, and the rest were selected to construct the testing dataset.

### Quantum mechanics (QM) calculations

All of the QM calculations were performed under the same protocol as that in the development of furanose-specific GLYCAM force field (ref) with the Gaussian 16 software package^31^ to maintain equal comparisons. Structural optimizations were performed at the HF/6-31G* level of theory, with five ring torsion angles and all *exo*-cyclic torsion angles restrained (Fig. 1). The electrostatic potentials were calculated on these optimized structures at the B3LYP/cc-pVTZ level of theory. The atomic partial charges were derived by employing the restrained electrostatic potential (RESP) charge fitting methodology with a weak hyperbolic charge restraint weight of 0.0005.^3,32^ The charge values for aliphatic hydrogen atoms were assigned to 0 by following GLYCAM force field parameter development philosophy.^19^

### Atom type symmetry function

ATSF was constructed under the framework of ACSF, whose coordinates were calculated from cartesian coordinates of atoms.^18^ The cutoff function in ATSF was that same as that in ACSF:3
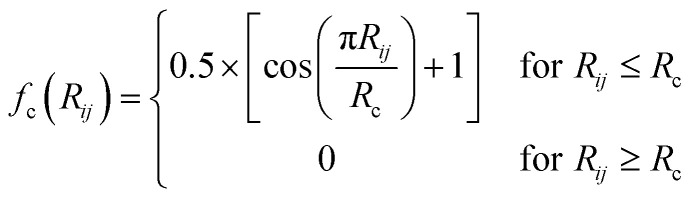
with *R*_c_ set to 99 Å to include all atoms for molecules included in this study.

Radial components of atom *i* were calculated *via* a sum of Gaussians,4
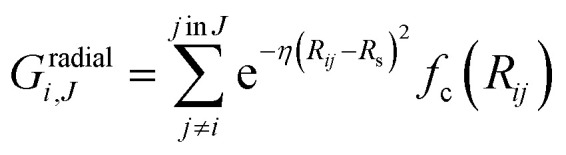
in which *R*_s_ and *η* are both set to 1.0. Atom *j* is an atom in atom types *J*. Please note that the atom *i* could be in atom type *J*. The assembly of *G*^radial^_i_ with different atom types constructs the radial components in ATSF for atom *i*.

Angular components for atom *i* were constructed as:5
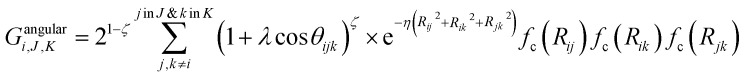
with the parameters of *λ* = 1.0, *ζ* = 1.0. Atom *j* and *k* are atoms in atom types *J* and *K*, respectively. The atom *i* could also be in atom types *J* and *K*. The assembly of *G*^angular^_i_ with different combinations of atom types constructs the angular components in ATSF for atom *i*. For each atom *i* in the molecule, the ATSF coordinates can be assembled as:6
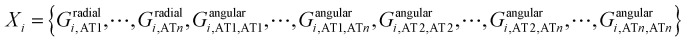
where AT stands for atom type.

The training and testing sets were generated by *S* = {(*X*_1_,*y*_1_), (*X*_2_,*y*_2_),⋯, (*X*_*n*_,*y*_*n*_)}, where *y*_1_, *y*_2_, …, *y*_*n*_ are the RESP derived charges for the corresponding atoms.

The charge predictions were achieved *via* multiple random forest regression (RFR) models, the sum of the predicted charges for an individual molecule is not necessarily 0. The corrections were achieved by spreading the discrepancy based on the standard derivations for RFR predictions. This procedure was adopted from ref. 33.

### Random forest regression

RFR model was trained for atoms in each element using the scikit-learn library (version 0.18.1)^34^ with the following parameters: number of trees = 200, maximum depth = 6, minimum number of samples to split = 6, and minimum number of samples in leaves = 6.

### Molecular dynamics (MD) simulations

The initial coordinates for furanosides 1–4 (α and β) were obtained from GLYCAM website (http://www.glycam.org). All systems were solvated with TIP3P water^35^ using a 12 Å buffer in a cubic box, using the LEaP module in the AMBER16 software package.^36^ Force field valence parameters were taken from furanose-specific parameters in GLYCAM.^3^ The energy minimizations for these solvated furanoses were performed separately under NVT condition (500 steps steepest descent, followed by 24 500 steps of conjugate-gradient minimization). Subsequently, each system was heated to 300 K over a period of 50 ps, followed by equilibration at 300 K for a further 0.5 ns using NPT condition, with the Berendsen thermostat and barostat^37^ for temperature and pressure control, respectively. SHAKE algorithm^38^ was employed to constrain all covalent bonds involving hydrogen atoms, allowing a simulation time step of 2 fs throughout the simulations. After the equilibration, production simulations were carried out with the GPU implementation^39^ of the PMEMD.MPI module and trajectory frames collected at every 1 ps from the total of 300 ns. A non-bonded cut-off of 8 Å was applied to van der Waals interactions, with long-range electrostatics treated with the particle mesh Ewald approximation.

### Hydration free energy and protein–carbohydrate interaction energy calculations

Hydration free energies for 1–4 (α and β) and protein–carbohydrate interaction energies for 3 ATP-binding cassette (ABC) transporters with furanoses as ligands (PDB ID: 2VK2, α-d-Gal*f*-OH and α-d-Gal*f*-OH as the ligands; PDB ID: 3KSM, β-d-Rib*f*-OH as the ligand) were calculated with molecular mechanics-generalized born surface area (MM-GBSA) method using single trajectory approach. Monosaccharides in all systems were taken as the ligand in the calculation. Solvent molecules and proteins were taken as the receptors in hydration free energy and protein–carbohydrate interaction calculations, respectively. Each MM-GBSA calculation was performed on the 10 000 evenly extracted structures of each solvated system with 3 different charge sets: RESP charges individually derived from QM calculations, predicted charges by RFR models with ATSF, and the ensemble-averaged atomic charges from GLYCAM force field.

## Results and discussion

### Different atom categorizing schemes in symmetry functions

**Fig. 3:**
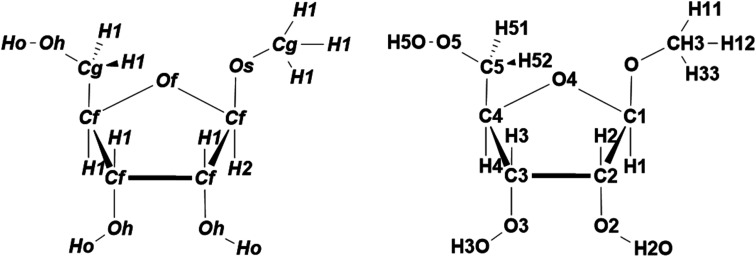
Atom types (left) and atom names (right) for furanosides in GLYCAM force field. In GLYCAM force field,^3^ “Cf” and “Cg” are for *endo*- and *exo*-cyclic carbon atoms, respectively. “Of” stands for the *endo*-cyclic oxygen atoms; “Os” and “Oh” are for *exo*-cyclic oxygen atoms in ether and hydroxyl groups, respectively. “H1” and “H2” stand for hydrogen atoms attached to a carbon atom that is bonded with one and two electron-withdrawing atoms, respectively; “Ho” is for the hydrogen atoms in hydroxyl groups.

ATSF in the present study employed atom types defined in furanose-specific GLYCAM force field^3^ (Fig. 3) to divide atoms into more groups beyond chemical elements. When categorizing atoms by only three chemical elements, ACSF contains 9 coordinates. The size of ATSF increased to 41 coordinates when categorizing atoms by a total of eight different atom types. The descriptor needs to be sufficiently large to ensure an unambiguous distinction of different conformations.^16^ In addition, categorizing atoms by their atom types introduced structural perceptions to the descriptor by adding information of property or connectivity for atoms. For complete comparisons, the most subtle categorizing scheme was also employed to generate atom name symmetry function (ANSF), where atoms were divided by their atom names (Fig. 3) and each single atom was in a unique category (Fig. 3). The size of ANSF for furanoside was dramatically increased to 276 coordinates and undoubtedly made this scheme unpractical for efficient calculations. Remarkably, ANSF abolished all chemical or structural information from the descriptor.

### Performances for atom type symmetry function

The performances of ATSF, ACSF, and ANSF were, firstly, evaluated individually by comparing the predicted charge values to the corresponding expected values, aka RESP values.^32^

**Fig. 4:**
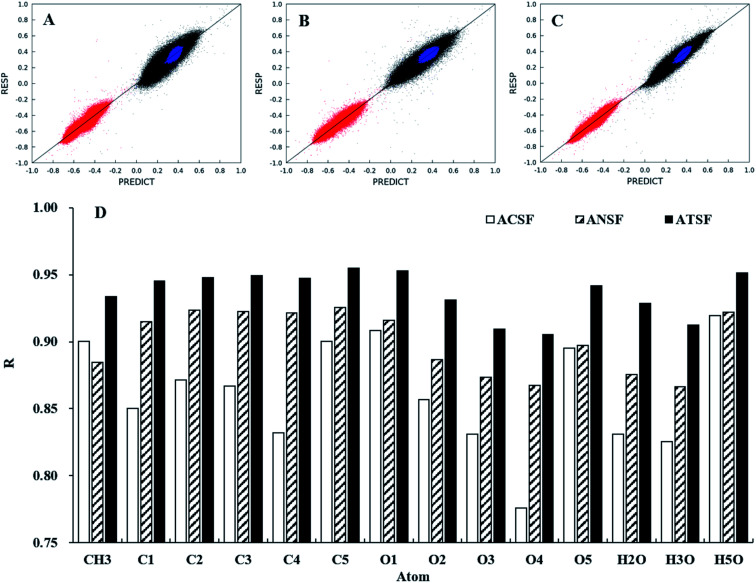
Comparisons for predicted and the corresponding RESP charges in 1–4 (α and β). Predictions of the CA charges for carbon (black), oxygen (red), and hydrogen (blue) atoms were performed under trained RFR models with ACSF (A), ANSF (B), and ATSF (C). The predictions were evaluated with their Pearson coefficients for different atoms (D).

Data in panel C of Fig. 4 appeared to be less scattered than those in panel B and C, which suggested a better correlation achieved by ATSF than ACSF and ANSF. The Pearson correlation coefficients for the CA charges predicted with ATSF reference to RESP charges derived from QM calculations increased averagely by 9% and 4%, respectively, comparing to ACSF and ANSF. The Pearson coefficients for all atoms were larger than 0.9 for those predicted with ATSF and systematically higher than the other two descriptors. This stronger correlation indicated RFR models with ATSF were able to produce more accurate CA charges than ACSF and ANSF. The results of linear fittings between predicted and RESP-fit charges were shown in Table 1. The slopes and intercepts to *Y*-axis for predictions with ATSF were close to 1 and 0, respectively, implied charges predicted values were not overestimated or underestimated. It is worth noting that RFR models with ANSF, which has a substantially larger size, did not produce higher quality predictions comparing to ATSF. This suggested that the chemical perceptions in categorizing scheme is crucial and increasing the size of coordinates without chemical perceptions would not necessarily guarantee more accurate predictions.

**Table 1:** Linear fits^a^ results between atomic charges predicted with different descriptors and their corresponding RESP charges derived from QM calculations

Atom	ACSF		ANSF		ATSF	
	*a*	*b*	*a*	*b*	*a*	*b*
CH3	1.038	−0.008	0.902	0.021	1.042	−0.009
C1	1.054	−0.020	0.931	0.026	1.047	−0.017
C2	1.068	−0.018	0.935	0.016	1.043	−0.011
C3	1.071	−0.017	0.930	0.017	1.055	−0.013
C4	1.077	−0.017	0.938	0.014	1.081	−0.018
C5	1.077	−0.017	0.934	0.015	1.034	−0.008
O1	1.036	0.014	0.924	−0.030	1.040	0.016
O2	1.063	0.038	0.907	−0.057	1.066	0.040
O3	1.096	0.059	0.886	−0.070	1.087	0.053
O4	1.151	0.065	0.886	−0.049	1.119	0.052
O5	1.051	0.030	0.911	−0.053	1.053	0.031
H2O	1.081	−0.030	0.900	0.038	1.062	−0.023
H3O	1.075	−0.027	0.882	0.045	1.087	−0.033
H5O	1.051	−0.019	0.927	0.027	1.035	−0.013

a Linear fit was achieved by *y* = *a* × *x* + *b*.

RFR models with ATSF have shown great potentials in predicting atomic partial charge values from different conformations of furanosides. It is also essential to demonstrate their advances in representing the electrostatic potentials of furanosides by reproducing molecular dipole moments,^40–44^ which is the first order of multipole expansions of electrostatic potentials and strongly depend on the conformation of the molecule. The average absolute differences of dipole moments between QM calculated values and the corresponding MM calculated values with different charge models were shown in Table 2. The differences of dipole moments computed from atomic partial charges predicted with ATSF were 43% and 48% smaller than those computed with ACSF and ANSF, respectively. It is worth noting that RESP charge models has the lowest dipole moment differences, which suggested that this charge model is appropriate to represent the electrostatic potential variations for furanosides and be utilized as the references for RFR model training.

**Table 2:** Average absolute differences between QM calculated dipole moments and the corresponding MM calculated values with different charge models for all conformations of furanosides in the testing and training data set

Charges	RFR models with			RESP
	ACSF	ANSF	ATSF	
〈|Dipole difference|〉^a^	0.28 ± 0.23	0.31 ± 0.32	0.16 ± 0.16	0.04 ± 0.03

a In Debye (D).

So far, RFR models with ATSF demonstrated their capabilities of predicting atomic partial charges with QM quality. To further confirm the validities of ATSF and its CA charges, the electrostatic-related interactions, carbohydrate–water and carbohydrate–protein interactions, for furanoses computed with predicted charges were compared to those with RESP charges.

### Performances on carbohydrate–water interaction energy calculations

Carbohydrate–water interactions, quantified by their hydration free energies, substantially depend on their electrostatic interactions. Thus, the quality of the atomic charges can be evaluated by their computed hydration free energies.^33,45^ In terms of conformation adaptive charges, the validity can be tested by comparing their computed hydration free energies to those computed with individual RESP-fit charges. Moreover, the hydration free energy from a single solute conformation could introduce errors.^46^ So, the averaged hydration free energies computed with conformational adaptive, RESP-fit, and ensemble-averaged charge sets for 100 000 structures of each furanoside in 1–4 (α and β) extracted from explicit solvent MD simulations were compared (Table 3). The hydration free energies calculated with CA charges predicted with ATSF are comparable to those computed with RESP-fit charges. The difference is 0.4 kcal mol^−1^, which is significantly less than that computed from ensemble-averaged charge sets (1.0 kcal mol^−1^). The differences among these calculated hydration free energies are significant (*p*-value < 0.0001), because of the large amount of structures employed in hydration free energy calculations, although the standard deviations are mostly over 3.0 kcal mol^−1^.

**Table 3:** Hydration free energies for 1–4 (both α and β) computed with different atomic partial charge sets

	Ensemble-averaged		RFR model with ATSF		QM derived RESP	
	Average	Stdev	Average	Stdev	Average	Stdev
1α	−6.8	3.2	−8.2	3.2	−7.8	3.4
1β	−6.9	3.2	−7.4	3.2	−8.0	3.3
2α	−6.8	3.1	−7.9	3.1	−7.8	3.2
2β	−6.5	3.0	−8.2	2.9	−7.6	3.1
3α	−6.7	3.1	−7.0	3.1	−7.9	3.3
3β	−6.7	3.1	−7.2	3.1	−7.4	3.2
4α	−7.1	3.2	−8.5	3.2	−8.3	3.3
4β	−6.9	3.2	−7.3	3.1	−7.9	3.4
〈|Difference|〉	1.0		0.4			

It is worth noting that the hydration free energies do not include the entropic penalties, therefore, the values may be more negative than the experimental measured values.

### Performance on carbohydrate–protein interaction energy calculations

Hydrogen bonding interaction is one of most popular and crucial hydrophilic interactions between carbohydrate molecules and proteins,^47–49^ due to the richness of hydroxyl groups presence in the *exo*-cyclic moieties. Accurately representing electrostatic potentials of carbohydrate molecules is crucial for correctly modeling the strength of hydrogen bonds between carbohydrate molecules and proteins. Yet, the static charge model lacks the accuracy for representing the electrostatic variations due to the changes from both *endo*- and *exo*-cyclic conformations of furanoses while interacting with proteins. Thus, the CA charge sets could improve the accuracy for carbohydrate–protein interaction energy calculations.

The computed MM-GBSA energies for three ABC transporter complexes with furanoses as ligands were listed in Table 4. The 〈|error|〉 for MM-GBSA energies with CA charges predicted by RFR models with ATSF, comparing to that calculated with RESP charges derived from QM calculations, was only 0.3 kcal mol^−1^, while that with the ensemble-averaged charges was 0.9 kcal mol^−1^.

**Table 4:** MM-GBSA energies for three ABC proteins computed with different atomic partial charge sets

Ligands	QM derived RESP		RFR model with ATSF		Ensemble-averaged	
	Average	Stdev	Average	Stdev	Average	Stdev
α-d-Gal*f*-OH	−31.7	1.9	−31.3	2.2	−31.9	2.3
β-d-Gal*f*-OH	−26.8	2.0	−27.0	2.4	−27.9	2.1
β-d-Rib*f*-OH	−19.9	2.1	−19.6	2.3	−21.2	2.4
〈|Error|〉			0.3		0.9	

The CA charge set predicted by RFR models with ATSF, comparing to the static charge model, showed improvements for including the electrostatic potential variations seen by individually derived charge values from QM calculations in their dynamic charge values. Similar to hydration free energies, these values do not include the entropic penalties, therefore, do not reflect the results measured from experiments.

## Conclusions

Atom type symmetry function (ATSF) categorized atoms by their atom types defined by the properties and connectivity of atoms in MM force field, beyond chemical elements in ACSF, and formed a more detailed structural description for furanoses that have complicated conformational spaces but limited chemical elements. Hence, the RFR models with ATSF produced more accurate predictions of CA charges and dipole moments than those with ACSF, which suggested ATSF obtained improvements in representing structural information for furanoses. The CA charge predicted by RFR models with ATSF, comparing to the static ensemble-averaged charges, employed in computing carbohydrate–water and carbohydrate–protein interaction energies showed a better agreement to those computed with individually derived RESP charges, which also demonstrated that CA charges were able to include the electrostatic potentials variations into the dynamic charge values.

Improvements achieved by ATSF in representing structural information for furanoses suggested that introducing structural perceptions to the descriptor and increasing the size of the coordinates could improve the performance of ACSF in describing furanoses. Furthermore, ATSF outperforming ANSF that had a significant larger size of coordinates but removed all chemical or structural perceptions of atoms suggested that categorizing atoms by atom types generated a suitable size of coordinates that represented the key structural features for furanoses. Additionally, this categorizing scheme endued ATSF with the exceeding potent transferability to other biomolecules thanks to the broad implementations of MM force fields for biomolecules.

## Conflicts of interest

There are no conflicts to declare.

## Supplementary Material
